# Contextualizing the results of an integrative review on the characteristics of dementia-friendly hospitals: a workshop with professional dementia experts

**DOI:** 10.1186/s12877-023-04312-3

**Published:** 2023-10-19

**Authors:** Christina Manietta, Daniel Purwins, Anneke Reinhard, Melanie Feige, Christiane Knecht, Birgit Alpers, Martina Roes

**Affiliations:** 1 Deutsches Zentrum Für Neurodegenerative Erkrankungen (DZNE), Witten, Germany; 2 Faculty of Health, School of Nursing Science, Witten/Herdecke University, Witten, Germany; 3 University Medical Center Hamburg-Eppendorf, Hamburg, Germany; 4 FH Münster University of Applied Sciences, Münster, Germany

**Keywords:** Cognitive impairment, Involvement research, Review, Acute care, Dementia-sensitive

## Abstract

**Background:**

To become a dementia-friendly hospital (DFH) is increasingly being discussed in health care practice, research, politics and society. In our previous integrative review, we identified six characteristics of DFHs. To thoroughly discuss and contextualize these characteristics in relation to hospitals in Germany, we involved professional dementia experts in our review process.

**Methods:**

At the end of our review process, we involved professional dementia experts at the ‘contributing’ level of the ACTIVE framework to discuss and reflect on the six DFH characteristics we identified. We conducted a group process in the form of a one-day workshop. The workshop consisted of four steps: 1. presentation of review results (input), 2. modification of DFH characteristics and rating of their relevance in smaller working groups, 3. discussion of group results in plenary and 4. questionnaire for prioritization and rating of feasibility. The data were analyzed in MAXQDA using content analysis and descriptive statistics.

**Results:**

A total of 16 professional dementia experts working in hospitals participated in the workshop. All the previously identified characteristics of a DFH were rated as relevant or very relevant for patients with dementia, their relatives and health care professionals from the professional dementia experts’ perspective. They made a few modifications of the six characteristics at the level of subcategories, aspects, and descriptions. The feasibility of the characteristics in hospitals was critically discussed regarding resources, hospital structures and processes, the role of nurses, and the current care situation of people with dementia in hospitals. More than half of the subcategories of the characteristics were considered very difficult or difficult to implement by most professional dementia experts.

**Conclusion:**

The involvement of professional dementia experts helped us contextualize our review findings within the German hospital setting. These results highlight the need to consider resources, funding options, influencing factors, and the current situation and culture of care provided by hospitals before implementing DFH characteristics. Beside the involvement of professional dementia experts and various health care professionals, the involvement of other stakeholders, such as people with dementia and their relatives, is necessary in future research for the development of a DFH.

**Supplementary Information:**

The online version contains supplementary material available at 10.1186/s12877-023-04312-3.

## Background

Hospital stays are often a burden for people with dementia and are associated with negative consequences. People with dementia are at high risk of hospitalization-related functional decline, delirium, falls, mortality, longer hospital stay and nursing home admission [1–3]. The hospitalization of people with dementia is also a challenge for the various health care professionals working in hospitals. Health care professionals face a dilemma: on the one hand, they are confronted with existing structures, closely timed procedures and lack of resources; on the other hand, they are caring for patients who require person-centered care and have complex needs that are not met by the existing system [4–6].

There are various efforts and strategies to improve the care of people with dementia in hospitals [7–10]. In this context, a dementia-friendly hospital (DFH) is increasingly being discussed in health care practice, research, politics and society [11–16]. The aim of our DEMfriendlyHospital study is to identify the characteristics of dementia-friendly hospitals based on an integrative review and interviews with patients with dementia, their relatives and professional dementia experts from various health care professions. In our integrative review, we identified the following six characteristics of a DFH: *continuity*, *person-centeredness, consideration of phenomena within dementia, environment*, *valuing relatives* and *knowledge and expertise* within the hospital [17].

To contextualize these six characteristics of DFHs identified in our integrative review, we involved professional dementia experts from a hospital as stakeholders in the last step of our review process. The involvement of stakeholders such as patients, caregivers, family members, and professional or academic experts in reviews is increasingly reported [18]. Involving stakeholders in one or different stages of the review process, such as development of the research question, conduct of the review, interpretation and dissemination of the results, is used to improve its quality, relevance and impact on health practices [18–21].

However, a scoping review by Pollock et al. [18] found that the quality of reporting the involvement of stakeholders in reviews is very poor, and only 32 of 291 included reviews comprehensively reported the methods of involvement. In this article, therefore, we describe the involvement of professional dementia experts in our integrative review and reflect on the conducted methodological procedure, in addition to the content component related to DFHs.

## Aim

The aim of stakeholder involvement was to discuss and reflect the results of our integrative review of DFHs with professional dementia experts. We focused on obtaining their views regarding the content and feasibility of the DFH characteristics. This allows us to contextualize the results of our review in relation to hospitals in Germany. According to this aim, we developed the following research questions:

- How do professional dementia experts rate the relevance of the six identified DFH characteristics?
- What modifications are needed from the point of view of professional dementia experts?
- Which characteristics are rated most important by professional dementia experts?
- How do professional dementia experts rate the feasibility of these DFH characteristics in hospitals in Germany?

## Methods

We involved stakeholders at the ‘contributing’ level of the *Authors and Consumers Together Impacting on eVidencE* (ACTIVE) framework [20, 21] at the end of the review process to discuss and reflect on our completed results with the help of the views, opinions and experiences of the professional dementia experts. To involve the professional dementia experts, we conducted a group process [20] in the form of a workshop. The results of this workshop did not directly influence the results of our integrative review but were considered an independent result and will be used for the next steps in our DEMfriendlyHospital study. These steps include interviews with patients with dementia, their relatives and professional dementia experts from various health care professions. In the final step of our study all results will be synthesized in a framework of DFH.

The method and the results of our integrative review are described in detail elsewhere [17] (a brief description of the DFH characteristics, including the subcategories, is described in Table 1).

**Table 1:** Description of DFH characteristics including their subcategories [17]

**Characteristics** Subcategories	**Description**
**Continuity**	Continuity is created for both the patient with dementia and their care during and after the hospital stay**.** Continuity is characterized by staff, location, daily structure, companionship, being informed, planning in advance and crossing sector boundaries
Staff	Continuity of staff is characterized by same staff, a small group of staff and a permanent professional contact person. This helps to build a relationship with the patient. Furthermore, this provides the feeling of continuity for the patient and supports continuity of their care
Location	Continuity of the location is characterized by service coming to the patient and avoiding internal transitions
Daily structure	Daily structure creates continuity for the patients. They are supported in structuring their day, and the hospital procedures are tailored to their individual daily rhythm and structure
Companionship	Continuity is created for the patient by the company of people who are as familiar with them as possible. Someone is close to the patient on the ward so that the patient does not feel alone. The patient is also offered activities in the company of others and is escorted outside the ward
Being informed	Continuity for the patient and their care is ensured by all professionals involved having the information about the patient that is necessary for their care during the hospital stay and discharge process as well for the post-acute care phase. If the patient is unable to provide the information themselves, sharing information with third parties such as relatives and internal and external health professionals is essential. The information needed to provide care includes general information (e.g., the patient’s condition, symptoms, diseases, care, treatment, medication) and dementia-related information (e.g., dementia diagnosis, behavior)
Planning in advance	Planning in advance means that the patient’s care is planned in advance related to admission, during the hospital stay and discharge to ensure continuity of care
Crossing sector boundaries	Crossing sector boundaries is characterized by working together and networking with other health care providers especially pre- and posthospital providers to achieve continuity of care during and after the hospital stay
**Person-centeredness**	Person-centeredness is characterized by knowing the person with dementia, the positive attitude exhibited toward the person with dementia and caring for the individual in a person-centered way
Knowing the person	Knowing or getting to know the person with dementia involves acquiring the usual information collected during the hospital stay (e.g., diagnoses or medical history) but also knowing the person beyond that, for example having information about their behavior, preferences, habits, biography and relatives Knowing the person is important since it influences the attitudes toward the person and builds a basis for a person-centered care
Attitude toward the person	The attitude of the staff toward the person with dementia (and their relatives*) is characterized by “seeing the person” behind the diagnosis, empathy, respect and appreciation*
Caring for the person	Caring for the patient in a person-centered way is characterized by fostering a personal relationship with the patient, respect for and promotion of their autonomy as well as care tailored to the person To provide care in a person-centered way, knowing the person and having a positive attitude toward the person are essential prerequisites
**Consideration of phenomena within dementia**	Dementia and its consequences (e.g. impact on everyday living) but also other phenomena and risks relevant to the care provided, are considered within the context of dementia. The focus of care is thus not only on the acute health issue and the primary reason for hospitalization but also on dementia
What? Phenomena	Considering dementia-specific symptoms, as well as particularly other care phenomena and risks in context of dementia during hospitalization
How? Methods	The phenomena are considered during the hospital stay (if possible) by identifying, diagnosing, preventing, treating and taking care of them
**Environment**	The environment supports the patient in orientation, activation, and independence and provides safety, creates familiarity and calm
Orientation	Temporal, local and situational orientation of the patient is promoted by the environment
Activation	The environment promotes patient activation in the form of movement, social interaction, and independent engagement with the environment
Familiar	The environment creates familiarity for the patient through familiar persons, personal items, homely design, and customizable interiors
Calm	The environment conveys calm to the patient by reducing environmental stimuli and providing comfort
Independence and safety	The environment promotes the patient’s independence while providing safety through measures that enable or limit access and various aids
**Valuing relatives**	Valuing relatives (relatives are defined here as persons who are close to the patient with dementia, i.e., they can be family members but also friends and neighbors) is characterized by a welcoming culture for relatives (always welcome), recognition of relatives as partners and experts, their involvement during the patient’s hospital stay, and taking care of them
Always welcome	Relatives are always welcome in the hospital. They can be with the patient at any time around the clock. The welcoming culture is also reflected in the hospital’s structures and services
Recognition	Recognizing relatives as experts due to their experience and as partners in the patient’s care
Involvement	Relatives are enabled to be involved during the patient’s hospital stay in different ways related to information (receiving and providing information), mediation between patient and hospital staff, care (active and passive) and decision-making. Involvement of relatives is supported by the welcoming culture. The degree of involvement considers the patient’s wishes and the wishes, burdens and capabilities of the relatives
Taking care	Relatives are also taken care of by recognizing and considering their needs, as well as their burden, and offering them tailored support
**Knowledge and expertise**	The complex care of patients with dementia in the hospital requires different knowledge and expertise related to dementia and various professions and disciplines within the hospital
Dementia-specific	Dementia-specific knowledge and expertise is available at different levels. All staff have a basic knowledge of dementia. In addition, there are dementia or geriatric experts who can be involved in the care of patients with dementia and support the staff
Multiprofessional	Multiprofessional knowledge and expertise is available for the care of patients with dementia. Therefore, professionals from diverse disciplines are involved in care and different ways of working together are used to bundle the different expertise and knowledge and enable a change of perspective and thinking, whereby the care can be applied in a more holistic manner

### Recruitment of participants

Participants for the workshop were recruited from a network of dementia experts at a cooperating university hospital in a major city in Germany. The network consists of nurses who have completed a nine-day training program on “the older patient with cognitive impairment”, which is provided by the university hospital to internal and external health care professionals. After completing this training, the dementia experts work in the hospital according to their professional qualifications across all departments and function as multipliers of dementia-related knowledge to promote awareness of people with dementia in the hospital and to improve their care. The network of dementia experts meets three times a year. The professional dementia experts who attended our workshop were recruited via the network coordinator. She distributed the invitation for the workshop to all dementia experts who were part of the network. The only two inclusion criteria for participation were that the dementia experts were members of the network and would have at least started the nine-day training. The workshop took place at one of the regular network meetings in September 2021.

### Description of the workshop procedures

**Fig. 1:**
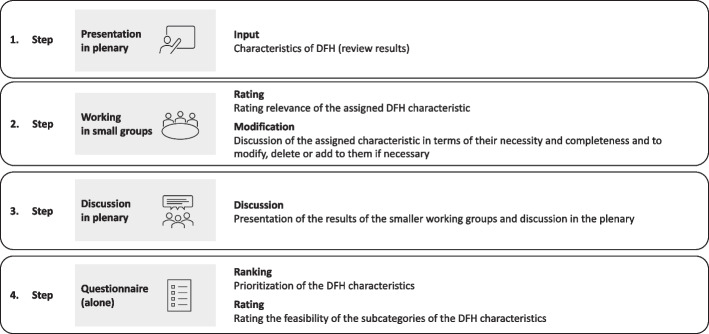
Steps of the workshop

The workshop lasted seven hours and consisted of four steps (Fig. 1).

- Step 1: We started with a short presentation (CM, MR) of the key findings of the review [17] (i.e., mind map of the six characteristics of DFH) and details about the workshop (e.g., aim, process, tasks).
- Step 2: The group was then divided into six smaller working groups of 2 to 4 dementia experts. Each group was assigned a different characteristic of the previously identified six DFH characteristics.

All groups were given the same three tasks:

- First, to discuss and rate the relevance of the assigned DFH characteristic for the patients with dementia, their relatives and the health care professionals in the group and to obtain reasons for their ratings. The ratings were made on a 4-point Likert scale (1 = “not at all relevant”, 2 = “less relevant”, 3 = “somewhat relevant”, 4 = “very relevant”).
- Second, to discuss each subcategory of the assigned DFH characteristic and its contents (aspects and descriptions) (Table 1) in terms of their necessity and completeness, and to modify, delete or add to them if necessary.
- Third, to discuss whether the assigned DFH characteristic with its subcategories is presented correctly in its entirety and, if necessary, to add to or modify it.

Each group received a mind map of all DFH characteristics, one poster per task and additional working materials on the assigned characteristic (task 1: characteristic definition, task 2: descriptions of the subcategories, their subordinate aspects and their detailed descriptions (Table 2), task 3: names of all subcategories on cards) and a detailed description of the tasks. To support tasks 2 and 3, the questions “What is (not) necessary?” and “What is missing?” were written on the posters.

**Table 2:** Structure of the characteristic on the example of “continuity” [17]

Characteristic structure	Description
**Characteristic:** **Continuity**	Continuity is created for both the patient with dementia and their care during and after the hospital stay**.** Continuity is characterized by staff, location, daily structure, companionship, being informed, planning in advance and crossing sector boundaries
**Subcategory:** ***Staff***	Continuity of staff is characterized by same staff, a small group of staff and a permanent professional contact person. This helps to build a relationship with the patient. Furthermore, this provides the feeling of continuity for the patient and supports continuity of care
Aspect: Same staff	The same staff are involved in the care of the patient, and changes in staff are avoided. This refers to the various professional groups (e.g., nursing staff, physicians, housekeeping, volunteers)

A time frame of 90 min was set for working on the three tasks. The researchers (CM, DP, AR, MR) were available for questions at any time during the processing time and visited the individual groups to clarify questions about the task or terminology, for example, but did not interfere in the discussion. The results of the discussion of the smaller working groups were written on posters.

- Step 3: The smaller working groups presented their results to the whole group in short presentations of no more than 20 min. Two of the researchers (CM, MR) moderated the discussion after each presentation. The presentations and discussion in plenary were written down in a protocol by two members of the research team (DP, AR). In addition, the posters were photographed to document the results.
- Step 4: The professional dementia experts prioritized the DFH characteristics and rated their feasibility in an anonymous questionnaire. The questionnaire was developed by two researchers (DP, CM) and reviewed, discussed and adapted by the other members of the research team (MR, AR) in a total of three team meetings. The participants were asked to rank the three most important characteristics for a DFH with numbers from 1 to 3. They were also asked to rate the feasibility of the subcategories of the characteristics using a 4-point Likert scale (1 = very difficult to implement, 2 = difficult to implement, 3 = easy to implement, 4 = very easy to implement) with the additional options “not feasible at all” and “I cannot judge”.

We also collected sociodemographic data such as age, gender and qualification of the professional dementia experts via a second anonymous questionnaire to describe the group of professional dementia experts. The participants were asked to complete both questionnaires after the workshop. The network coordinator collected the questionnaires within a few days and sent them to the research team.

### Analysis

The protocols and photos of the posters were analyzed in MAXQDA [22] using content analysis [23]. For this purpose, we used a mixed deductive-inductive approach [23]. The following categories were initially formed and derived from the research questions: *reasons for relevance* and *modification*. The text passages were then read line by line and assigned deductively to the categories. In the next step, the text passages of both categories were assigned deductively to subcategories. The text passages of the category *modification* were deductively assigned to the subcategories: *addition* (sub-subcategories: *subcategory added, aspect added, description contents added, description contents changed*), *deletion* (sub-subcategories: *aspect deleted, description contents deleted*), *renaming* (sub-subcategories: *subcategory renamed, aspect renamed*) or *merging* (sub-subcategories: *aspect merged*). For the category *reasons for relevance*, the text passages were assigned to the subcategories *people with dementia*, *relatives* and *health care professionals*. Reasons for relevance were differentiated per group by identifying inductively sub-subcategories from the data. These sub-subcategories were created directly at a higher level of abstraction (e.g., positive patient outcomes). In addition, the category *influencing factors* could be inductively identified from our data and finally included in our results. Similar to the deductive categories, the text passages were first assigned to the category *influencing factors* and in a second step, further differentiated by inductively identifying subcategories from the data. The subcategories were formulated with a lower level of abstraction and closer to the content (e.g., staff ratio) to describe the influencing factors in detail. The initial coding was conducted by one researcher (CM) and checked by the research team (DP, AR, MR). The demographic data, rankings (prioritization) and Likert scales (relevance, feasibility) were analyzed using descriptive statistics (i.e., frequencies, percentage, means and standard deviations).

## Results

### Description of the participants

A total of 16 participants took part in the workshop. All participants completed the nine-day dementia expert training program. Most participants had already been working as dementia experts for more than three years (*n* = 13), one participant had been working as a dementia expert for two to three years, and two participants had been working for less than one year. All participants were nurses and worked in different disciplines, such as surgery (*n* = 7), interdisciplinarity (*n* = 3), conservative medicine (e.g., internal medicine, neurology) (*n* = 2), psychiatry (*n* = 1), geriatrics (*n* = 1), stroke unit (*n* = 1) and anesthesia (*n* = 1). Nine participants had at least one additional qualification in addition to the dementia expert program, e.g., hygiene experts (*n* = 3), practice instructors (*n* = 3), specialist trainings in psychiatry (*n* = 1), anesthesia and intensive care (*n* = 1), palliative care (*n* = 1) or management (*n* = 1). Further characteristics of the participants are shown in Table 3.

**Table 3:** Characteristics of the participants (*N* = 16)

Variable	Category	n (%)
Gender	Female	13 (81.25%)
	Male	1 (6.25%)
	Missing data	2 (12.5%)
Age	20 to 29 years	1 (6.25%)
	30 to 39 years	5 (31.25%)
	40 to 49 years	1 (6.25%)
	50 to 59 years	6 (37.50%)
	60 years or older	1 (6.25%)
	Missing data	2 (12.5%)
Working hours	Fulltime	1 (6.25%)
	Parttime	15 (93.75%)

### Relevance of the six DFH characteristics

Five of the six DFH characteristics were rated as “very relevant” for the patient with dementia except for “valuing relatives”. From the perspective of the professional dementia experts, the characteristic “valuing relatives” was rated as “somewhat relevant” for the patients; the professional dementia experts reasoned that this characteristic may not be the highest priority for the patients. The relevance of the other characteristics for patients with dementia was justified by an improvement in hospital care and associated positive outcomes for them, such as a reduction in stress and an increase in well-being, orientation, feelings of safety, sense of respect, and an improvement or stabilization of their condition and dementia symptoms. Another rationale was that these DFH characteristics could contribute to improving dementia diagnostics as well as increasing patients’ knowledge and acceptance of their dementia diagnosis.

The professional dementia experts deemed all six characteristics of a DFH as “very relevant” with regard to the relatives of the hospitalized patient with dementia. This was justified with positive outcomes for the relatives, such as having their uncertainty and fears alleviated, believing the patient was in ‘good hands’, and creating a basis of trust. In addition, the professional dementia experts reasoned that the well-being of the relatives can be increased by a DFH, and this would have a positive effect on the patient. Furthermore, the high relevance was explained by the relief of relatives, the invitation to the relatives to communicate, the experience of being noticed and recognized for their care of the patient. It was also reasoned that characteristics such as “continuity” would help make the patient’s post-discharge condition more calculable for relatives. Moreover, the improved hospital structures for the relatives, such as contact persons and counseling and support offers, were mentioned in this context.

The professional dementia experts rated all characteristics except “valuing relatives” as “very relevant” with regard to the diverse health care professionals working in the hospital and rated “valuing relatives” as “somewhat relevant”. The reason behind this is that the patient should be the focus of attention from health care professionals. Furthermore, the impact on the patient and the responsibility of health care professionals (e.g., increase patient safety, provide professional care for people with dementia, and shorten the hospital stay) were reasons why the DFH characteristics were rated as relevant. DFH characteristics would also have an impact on the health care professionals and on their work, such as easing their workload, reducing their burden, improving multiprofessional collaboration or contributing to the professionalization of the nurses.

### Modification of DFH characteristics

Modifications were made at the level of subcategories, aspects, and their descriptions (Table 2). A detailed description of the modifications of the characteristics and the content of the discussion are presented in Table 4.

**Table 4:** Modification of the DFH characteristics and content of the discussion

Characteristics (incl*. subcategories,* aspects)	Modification	Content of discussion
**Continuity**		• The same level of knowledge for all health professions and time were described as prerequisites for the characteristic “continuity”. It was suggested that professionals other than nurses should also be trained in dementia care
***Staff***		
Same staff	Aspect deleted	• The same staff was proposed to be deleted, as this is not feasible in practice due to part-time workers and days off • A small team with a professional contact person was highlighted as particularly necessary • Several nurses should take over the care, who represent each other and know the patient and are no strangers to the patient with dementia
Small group of staff		
Professional contact person		
***Location***		
Service comes to the patient		• Location was highlighted in the discussion as particularly important for the patient with dementia • One participant said: “Any way that can be avoided is worth its weight in gold for people with dementia” • The feasibility of “service comes to the patient” was critically discussed. However, it was mentioned that this is already implemented in palliative care units • Avoiding emergency room stays was also critically discussed, since most patients with dementia are admitted via the emergency room
Avoiding internal transitions		
***Daily structure***		
Supporting daily structure		• Daily structure is considered important for the patient with dementia, but currently not feasible in practice
Tailored daily structure		
***Companionship***		
Being close by		• Companionship is considered important for the patient with dementia, but it depends on the individual patient • The feasibility of implementation depends on the staffing and whether this can be integrated into the daily routine of the ward, e.g., in surgery it is difficult to implement because functions are in the focus
Social activities		
Escort		
***Being informed***		
External		• Information exchange with external health care professionals is currently not seen as sufficient in practice, as the ambulance service hardly passes on any information and has too little contact with the patient with dementia
Internal		
***Planning in advance***		
Admission		• The professional dementia experts did not comment on this subcategory, and it was not discussed
During hospital stay		
Discharge		
***Crossing sector boundaries***		
Working together		• Crossing sector boundaries is important for the patient with dementia, but difficult to implement in practice due to staffing ratios and time • The professional dementia experts see more responsibility on the social workers/case managers for the cooperation with external health care providers, as they manage the discharge • Cooperation strongly dependent on individual motivation of staff, e.g., whether outpatient care is informed that patient transport is delayed • Regional networking would have to be carried out by a person responsible for this
Networking		
**Person-centeredness**		• It was described that a stronger consideration of the autonomy of dementia patients is missing in the current situation in hospitals and caring for the patients and themselves is necessary
***Knowing the person***		
Behavior		• It was highlighted in the discussion that “knowing the person” is important for the care of people with dementia • “Habits” and “preferences” were perceived as duplications and were merged • The content of “preferences” was partly perceived as duplicating those of the aspect “biography”, such as, for example, preferences related to activities and were deleted • The aspect “relatives” was renamed into another German word for relatives, which follows a broader understanding of relatives and therefore also includes other close persons such as friends and neighbors • The content of “relatives” was expanded to include not only knowledge about the social situation of the patient with dementia but also information such as occupation or social status of the relatives to be able to understand them better
Habits	Aspect merged	
Preferences	Aspect merged Description contents deleted	
Biography		
Relatives	Aspect renamed Description contents added	
***Attitude toward the person***	Aspect added	
Seeing the person		• It was highlighted that “attitude toward the person” should refer to all patients and not only to people with dementia and that this should be a general attitude of all health care professionals • The aspect “reflection in the team” was added. The reflection of the attitude and actions of the health care professionals on the team, e.g., in the form of case conferences, can contribute to a positive change in the attitude of health care professionals toward the person with dementia
Empathy		
Respect & appreciation		
***Caring for the person***		
Relationship		• The aspect “autonomy” was discussed as necessary but very difficult to facilitate due to the admission contract that is concluded with the hospital. It was mentioned that ensuring autonomy is very dependent on persons and situations • The aspect “tailored care” was also discussed as necessary but very difficult to facilitate due to the opportunities and resources of the ward
Autonomy		
Tailored care		
**Consideration of phenomena within dementia**		
		• Reflections were made on how to achieve consideration of phenomena within dementia and architecture, professionalism, training, staffing, the possibility of accommodating relatives, discharge management and care transition were mentioned in this context
***What? Phenomena***		
Dementia-specific symptoms	Description contents added	• Motor skills was added to the description of “dementia-specific symptoms” • Wandering was added to the description of “other(care) phenomena & risks”
Other (care) phenomena & risks	Description contents added	
***How? Methods***		
Identification & diagnostics		• The identification of cognitive impairment was highlighted in the discussion and described as central for a DFH • The professional dementia experts mentioned that dementia diagnostics are often not carried out in the hospital itself and the suspicion of cognitive impairment is not always passed on to further health care providers, as this may only be passed on by physicians • The professional dementia experts considered it useful that professional observation of phenomena in the context of dementia is divided between the different professional groups and that improved communication between the professional groups must be promoted for this purpose • The aspect of prevention was discussed in terms of practicability. For example, cognitive training is deleted, because it is difficult to implement in hospitals and not possible in every department
Prevention, treatment & care intervention	Description contents deleted	
**Environment**	Subcategory added	• The subcategory “social contacts/environment” was added because conversations and contacts with other people are important for patients with dementia to feel comfortable
***Orientation***		
Temporal orientation aids		• The professional dementia experts discussed orientation aids and mentioned that pictograms or color accents are important for orientation and that aids have to be individually adapted to the patient
Local & situational guidance		
***Activation***	Aspect added	
Spaces		• The subcategory “activation” was also highlighted as particularly important in the discussion • The aspects activity box or handbag, sufficient rollators, snack and drink stations were added
Activity items		
***Familiar***	Aspect added	
Familiar person		• The aspect of familiar “pictograms” for restrooms was added • The presence of familiar persons was discussed as particularly important, as well as the physical proximity of the relatives. The possibility of rooming-in was mentioned, as well as the special regulation during the COVID-19 pandemic to extend the visiting hours of people with dementia by physician’s order
Personal items		
Homelike designs		
Customized interior		
***Calm***	Aspect added	
Environmental stimulus		• Reduction to the necessary number of persons, e.g., no additional trainees or students, was added as an aspect
Comforts		
***Independence & safety***	Aspect added	
Access		• The aspect of “access to telephone/mobile phone” was added, so that the patients can contact their relatives at any time. The reason given was that this is particularly important for people with dementia since the COVID-19 pandemic and the associated visitor restrictions
Aids		
**Valuing relatives**	Subcategory added	• Related to this characteristic, it was added that it was necessary to find a consensus between hospital structures and the individuality of the patients with dementia and their relatives to ensure satisfaction on both sides • “Consideration of religion and culture” in relation to interactions, decision-making, treatment and care was added as a subcategory to the characteristic “valuing relatives”
***Always welcome***	Subcategory renamed Aspect added	• “Always welcome” was generally positive received, and it was suggested that this subcategory be renamed “welcoming culture” • The aspect of fixed consultation times for relatives was added to this subcategory, as it is currently difficult for relatives to obtain a consultation appointment with the attending physician and the physicians are thus taken more into responsibility • Regarding the aspect “visiting hours”, the professional dementia experts discussed the flexible visiting hours without restrictions and changed the description depending on individual assessment of how the patient with dementia reacts to the presence of the relatives • The professional dementia experts point out that a balance between the presence of the relatives is important to give them and the patient a rest and to “force” them to take time out through visiting hours • Regarding the “always welcome” policy about relatives, it was pointed out that consultation with fellow patients is necessary • The aspect of “rooming-in” was discussed critically regarding its practicability in acute admissions, although it is seen as very important especially in the acute phase. In addition, room occupancy problems were mentioned in this context, as a single room must be available for this purpose • The aspect of “room and interior” for relatives, e.g., retreat areas were seen as secondary and not feasible
Visiting hours	Description contents changed	
Rooming-in		
Room & interior		
***Recognition***		
As experts by experience	Description contents changed	• Recognition “as experts by experience” was critically discussed and the content of the description was changed, as this does not apply to all relatives. Accordingly, not all relatives have a good relationship with the patient, take over the care at home or live nearby, and it must be considered that some patients do not have relatives • Relatives should be valued, and the information from the relatives, who are experts by experience, is considered very valuable • It is not necessary to see relatives “as partners” in the care process during the hospitalization, in order not to put the relatives under pressure to feel obliged to take over the care in the hospital • Another argument against recognizing relatives “as partners” in the care process is the structured work of hospital staff versus the individual care and ideas of relatives • It was highlighted that relatives are partners in the discharge process and make an important contribution to it
As partners	Description contents changed	
***Involvement***		
Information	Description contents added	• The necessity of “information” was positively discussed, and information about medication and guardianship were added to the description • Ideas for practical implementation were discussed in the form of an information sheet that should be given to the relatives during admission • Involvement in “care”, such as accompanying patients to examinations, is also described as desirable, as it makes patients calmer, but this is not always possible to implement or plan for in acute phases • When involving relatives in personal care, it was pointed out that drains and wound dressings must be considered in this context • In description of “care”, monitoring was renamed to observe, perceive and report back • The involvement of relatives in day structuring was considered unrealistic, as this is not possible due to the hospital routine • The presence of relatives during operations was added to the description of “care” • Concerning involvement in “decisions”, the professional dementia experts mentioned that it should be noted that relatives do not always make decisions objectively and that they might lack the expertise to be involved in decisions, e.g., regarding the necessity of hospital admission • The description of “decisions” was supplemented with “living will” and “decisions related to resuscitation”. Additionally, “inclusion in care during hospitalization according to their wishes” was completed with “according to their wishes, views and life experience”
Mediation		
Care	Description contents added & changed	
Decision	Description contents added	
***Taking Care***		
Needs		• It was discussed as important to perceive the limits, needs and relief possibilities of relatives to assess and support the current care situation • The aspect “support” was judged by the professional dementia experts as not feasible in terms of time and modified with the reference to external counseling centers
Support	Description contents changed	
**Knowledge and expertise**		
***Dementia-specific***		
Basic knowledge		• It was discussed, that “dementia-specific” knowledge and expertise is not seen as relevant by most of the health care professionals due to a lack of awareness of people with dementia in most disciplines • All professional dementia experts agreed that “basic knowledge” among all hospital staff is needed, especially for service staff, to develop understanding of the patient group and to be able to respond to the patients accordingly • In this context, it was considered how the information about patients with dementia can be shared with service staff so that they are aware of dementia • The professional dementia experts called for more dementia-specific knowledge in the nurses’ education and in general among other health care professionals, e.g., physicians and therapists
Experts		
***Multiprofessional***		
Involvement	Description contents added	• Multiprofessional was highlighted as desirable in the discussion • The description of “involvement” was added to that of the nursing aides and the geriatric specialists • The need for “working together” in the care of patients with dementia was emphasized, as well as the need to clarify the responsibilities of the various health care professions
Working together		

#### Modification of subcategories

All subcategories of the characteristics were seen as necessary for a DFH, and no deletions were made at this level. New subcategories were added to two of the six characteristics by the dementia experts. The subcategory “social contacts/social environment” was added to the characteristic “environment”, reasoning that conversations and contacts with other people are important for the patient to feel comfortable. “Consideration of religion and culture” was added to the characteristic “valuing relatives”. The original subcategory “always welcome” of the characteristic “valuing relatives” was renamed “welcoming culture” because the presence of relatives should be assessed individually, depending on the patient’s reaction, the burden on the relatives and the other patients.

#### Modification of the aspects describing subcategories

Some aspects of subcategories were deleted (*n* = 1), added (*n* = 6), renamed (*n* = 1) or merged (*n* = 2). In the subcategory “staff” (characteristic: “continuity”), the aspect “same staff” was proposed to be deleted, as according to the professional dementia experts, this is not feasible in health care practice. Rather, several nurses, who substitute for each other and know the patient should care for him or her. Additionally, in several subcategories, aspects were added, such as, for example, in the subcategory “independence and safety” (characteristic: “environment”), the aspect of “access to telephone/mobile phone” was added so that the patients could contact their relatives at any time. Especially since the outbreak of COVID-19 and the associated visiting restrictions, this was particularly important for people with dementia according to professional dementia experts. Moreover, the aspect “relatives” in the subcategory “knowing the person” (characteristic: “person-centeredness”) was given another German word for relatives, which includes other close persons such as friends and neighbors in a broader definition of “relatives”. The aspects “habits” and “preferences” of the subcategory “knowing the person” (characteristic: “person-centeredness”) were perceived as duplications and were merged.

Some aspect descriptions were modified by deleting (*n* = 2), adding (*n* = 7), or changing (*n* = 5) content. For example, cognitive training in the aspect “prevention, treatment & care intervention” (subcategory: “How? Method”, characteristic: “consideration of phenomena within dementia”) was deleted because, according to the dementia experts, it cannot be implemented in all departments in hospitals. The description of the aspect “relatives” (subcategory: “knowing the person”, characteristic: “person-centeredness”) was expanded to include that not only knowledge about the social situation of the patient but also information such as occupation or social status of the relatives is necessary to be able to understand them better. The professional dementia experts added to the description of “involvement” (subcategory: “multiprofessional”, characteristic: “knowledge and expertise”) the involvement of nursing aides and specialized nurses for geriatrics. The description of the aspect “as a partner” (subcategory: “recognition”, characteristic: “valuing relatives”) was suggested to be changed, as relatives should not be seen as partners in the care process to prevent them from feeling obliged to take over care in the hospital. Another difficulty in seeing relatives as partners in the care process, according to the professional dementia experts, is that the structured work of hospital staff is opposed to the individual care and expectations of relatives.

### Prioritization of the six DFH characteristics

A total of 15 of the 16 professional dementia experts returned the questionnaires. Five of the 15 returned questionnaires were included in the analysis of prioritization of the DFH characteristics. The other questionnaires were not analyzable due to multiple answers. Four out of five dementia experts ranked the characteristic “knowledge and expertise” as the top priority, followed by “person-centeredness” (*n* = 3) and “continuity” (*n* = 3). The results of the ranking of the characteristics are shown in detail in Table 5.

**Table 5:** Ranking of the most important characteristics

Top	Characteristic (n)
**Top 1**	Knowledge and expertise (*n* = 4)
	Environment (*n* = 1)
**Top 2**	Person-centeredness (*n* = 3)
	Environment (*n* = 1)
	Consideration of phenomena within dementia (*n* = 1)
**Top 3**	Continuity (*n* = 3)
	Environment (*n* = 1)
	Person-centeredness (*n* = 1)

### Feasibility of the DFH characteristics in German hospitals

During the workshop, the feasibility of the characteristics was repeatedly mentioned by the professional dementia experts, and various influencing factors were described in the plenary discussion. For the implementation of the characteristics, time and staffing were seen as essential. In addition, rigid and absent structures and processes were described as barriers where nurses had limited spheres of influence. Lack of professionalization of nursing was mentioned in relation to the area of responsibility and the perception of nurses. The nurses feel that their profession is not valued by physicians. On the one hand, other health care professions (e.g., physicians) define the care of people with dementia and the consideration of dementia during hospitalization as a unique task of nurses. On the other hand, individual tasks are reserved for the physician. For example, sharing a suspected diagnosis and symptoms with other health care providers cannot be done by the nurses and is the sole responsibility of the physicians. Lack of knowledge, lack of interdisciplinary cooperation and agreements, the exclusive focus on the respective departments and care during hospitalization, and the lack of interest of physicians in the phenomenon of cognitive impairment were described as huge barriers.

Moreover, resources and the existing architecture, such as lack of rooms, were also mentioned as barriers. In addition, the increasingly shorter hospital stays and the mostly unplannable hospital admissions due to crises (e.g., falls) were described as negative factors influencing the implementation of the DFH characteristics. The personal commitment, interest and motivation of the individual health care professionals were listed as highly relevant facilitating factors.

All 15 returned questionnaires were included in the analysis of the feasibility rating of the DFH characteristics. None of the subcategories was assessed as “not feasible at all”. More than half of the subcategories (14 out of 23 subcategories) were rated difficult (dichotomous: very difficult/difficult) to implement by most professional dementia experts. Only the subcategories of the characteristic “valuing relatives” were considered easy (dichotomous: easy/very easy) to implement by most professional dementia experts except for “taking care”. The subcategories “taking care” (characteristic “valuing relatives”), “location” and “being informed” (both within characteristic: “continuity”), “dementia-specific” (characteristic: “knowledge and expertise”), and “attitude toward the person” and “caring for the person” (both within characteristic: “person-centeredness”) were rated as easy or difficult to implement by half or almost half of the professional dementia experts. A detailed presentation of the results is shown in Table 6.

**Table 6:** Rating of feasibility of the subcategories

**Subcategories**	**I cannot judge n (%)**	**Very difficult to implement** (1) **n (%)**	**Difficult to implement** (2) n (%)	**Easy to implement** (3) n (%)	**Very easy to implement** (4) n (%)	**Mean scores**	**Standard deviation**
**Continuity**							
Staff	1 (6.67%)	3 (20.0%)	10 (66.67%)	1 (6.67%)	0 (0%)	1.86	0.53
Location	1 (6.67%)	1 (6.67%)	6 (40.0%)	5 (33.33%)	2 (13.33%)	2.57	0.85
Daily structure	1 (6.67%)	2 (13.33%)	7 (46.67%)	5 (33.33%)	0 (0%)	2.21	0.70
Companionship	1 (6.67%)	2 (13.33%)	9 (60.0%)	3 (20.0%)	0 (0%)	2.07	0.62
Being informed	1 (6.67%)	1 (6.67%)	6 (40.0%)	7 (46.67%)	0 (0%)	2.43	0.65
Planning in advance	1 (6.67%)	2 (13.33%)	10 (66.67%)	2 (13.33%)	0 (0%)	2.00	0.55
Crossing sector boundaries	4 (26.67%)	1 (6.67%)	8 (53.33%)	2 (13.33%)	0 (0%)	2.09	0.54
**Person-centeredness**							
Knowing the person	0 (0%)	1 (6.67%)	8 (53.33%)	6 (40.0%)	0 (0%)	2.33	0.62
Attitude toward the person	0 (0%)	0 (0%)	8 (53.33%)	4 (26.67%)	3 (20.0%)	2.67	0.82
Caring for the person	0 (0%)	0 (0%)	8 (53.33%)	5 (33.33%)	2 (13.33%)	2.60	0.74
**Consideration of phenomena within dementia**							
What? Phenomena	3 (20.0%)	1 (6.67%)	7 (46.67%)	3 (20.0%)	0 (0%)	2.18	0.60
How? Methods	3 (20.0%)	1 (6.67%)	7 (46.67%)	2 (13.33%)	1 (6.67%)	2.27	0.79
**Environment**							
Orientation	0 (0%)	1 (6.67%)	10 (66.67%)	4 (26.67%)	0 (0%)	2.20	0.56
Activation	0 (0%)	1 (6.67%)	9 (60.0%)	5 (33.33%)	0 (0%)	2.27	0.59
Familiar	0 (0%)	4 (26.67%)	9 (60.0%)	2 (13.33%)	0 (0%)	1,87	0.64
Calm	0 (0%)	8 (53,33%)	3 (20.0%)	4 (26.67%)	0 (0%)	1.73	0.88
Independence & safety	0 (0%)	3 (20.0%)	8 (53.33%)	3 (20.0%)	0 (0%)	2.00	0.68
**Valuing relatives**							
Always welcome	0 (0%)	1 (6.67%)	4 (26.67%)	8 (53.33%)	2 (13.33%)	2.73	0.80
Recognition	0 (0%)	0 (0%)	3 (20.0%)	9 (60.0%)	3 (20.0%)	3.00	0.65
Involvement	0 (0%)	0 (0%)	3 (20.0%)	10 (66.67%)	2 (13.33%)	2.93	0.59
Taking Care	0 (0%)	2 (13.33%)	5 (33.33%)	6 (40.0%)	1 (6.67%)	2.43	0.85
**Knowledge and expertise**							
Dementia-specific	1 (6.67%)	0 (0%)	7 (46.67%)	6 (40.0%)	1 (6.67%)	2.57	0.65
Multiprofessional	1 (6.67%)	2 (13.33%)	8 (53.33%)	4 (26.67%)	0 (0%)	2.14	0.66

## Discussion

The involvement of professional dementia experts at the end of our review process allowed us to discuss and contextualize our findings using their views and experiences with hospitalized patients with dementia in Germany.

All six characteristics of a DFH that we identified in our previous review (*continuity*, *person-centeredness, consideration of phenomena within dementia, environment*, *valuing relatives* and *knowledge and expertise*) were judged by the professional dementia experts to be (very) relevant for the patients with dementia, their relatives and the health care professionals. The content of the characteristics essentially corresponded to their understanding of DFHs, and only a few modifications were needed. This might be related to the fact that the included descriptions of DFHs in our integrative review were primarily characterized by the perspective of professional dementia experts and health care practitioners [17]. Very little new content was added by the professional dementia experts. This could be due to the already comprehensive review results, which might have narrowed the view and resulted in fewer new aspects. To deepen the perspective of the professional dementia experts detached from the findings, interviews with multiprofessional dementia experts could be useful in the future to complete this perspective on a DFH.

Some subcategories of the characteristic “valuing relatives” were critically discussed by the professional dementia experts. They voiced the concern that phrases such as “always welcome” or “recognition” as a partner in the care process could be misunderstood and may send the wrong message. This could increase the feeling of pressure that their constant presence is required or that they should take over the care of their hospitalized family member. However, studies show that relatives perceive rigid visiting hours as a barrier for accompanying the patient with dementia [24] and that they get the impression that staff do not always welcome their presence [25]. Furthermore, relatives have uncertainties about their role and what is expected of them [24, 26]. In addition, they feel undervalued as a resource, that their concerns are not taken seriously, and that their expertise is not perceived by health care professionals [24, 26]. According to the study by Petry et al. [27], relatives want to play an active role in caring for the person with dementia in the hospital. Accordingly, it is important to involve not only professional dementia experts in the development of DFHs, but also people with dementia and their relatives.

The professional dementia experts discussed the feasibility of the characteristics and rated most subcategories as difficult to implement on average. The reasons given for this were primarily the current conditions in the hospitals, such as time and staffing, as well as structures and processes. These aspects on an organizational level are also described in other studies as barriers and challenges to caring for people with dementia in hospitals [4, 5, 28, 29].

Furthermore, the lack of statutory professional responsibility of nurses in Germany was mentioned as a barrier. On the one hand, dementia is seen primarily as a task for nurses; on the other hand, there is a lack of scope within the statutory responsibility and no recognition of their expertise by other health care professions (e.g., physicians). These findings are confirmed in a study by Pinkert et al. [5], in which nurses described a lack of support and recognition of their work by other health care professionals, as well as conflicts of competence with physicians in the context of caring for patients with dementia. Advancing tasks and statutory responsibilities of nurses [30] and implementing of dementia specialist nurses [31], which is common in other countries, could improve dementia care in hospitals in Germany. Nevertheless, the care of people with dementia in hospitals is a multiprofessional task, for which collaborative strategies such as multiprofessional case conferences [32, 33] or interprofessional education [34] as well as dementia-specific knowledge and awareness of all health care professionals are needed. The discussion with dementia experts and findings of other studies [4, 5, 25, 28, 35, 36] show that both are lacking. However, there are already best-practice approaches implemented in hospitals that address multiprofessional collaboration in the care of people with dementia, such as multiprofessional consultation teams [37].

The knowledge of staff is a key factor in providing quality care for people with dementia to appropriately address the abilities and needs of people with dementia [29]. Knowledge of staff also corresponds to the attitude of staff toward people with dementia and their stigmatization [29]. A study by Keogh et al. [38] shows that staff with prior dementia training are more likely to have positive attitudes toward people with dementia and higher perceived dementia knowledge.

Our findings highlight the need for a multifaceted implementation strategy tailored to the hospital [39–41] as well as the participatory involvement of different stakeholders [42] to enable implementation considering existing resources, influencing factors (barriers and facilitators) and the current care situation of people with dementia in the hospital.

### Lessons learned when involving dementia experts in reviews

In addition to contextualizing our review results within the German hospital setting, we were able to learn the following insights by involving professional dementia experts reflecting on our review findings:

- During the discussion, one challenge was to discuss the content of the DFH characteristics in detail, since the focus of the professional dementia experts was directed toward the practicability of the identified characteristics and their current situation in the hospital. This could be due to the already comprehensive description of the characteristics.
- The workshop required a lot of time for preparation (e.g., preparation of the results in a comprehensible language). We planned a one-day workshop, which in retrospect was too short for a detailed discussion of the comprehensive results of the review. The professional dementia experts commented that they were not used to this kind of theoretical or conceptual reflection and found that the tasks were too extensive and complex for one day.
- At the same time, the interest of the professional dementia experts in the topic was very high, which was experienced as very positive for the workshop, together with the atmosphere and the small working groups.

### Limitations

There are potential limitations that need to be considered. We only used a convenience sample of professional dementia experts as participants in our workshop. Due to COVID-19 pandemic restrictions, the possibility of including a broader range of stakeholders (such as people with dementia and their relatives) was not possible. In our ongoing DEMfriendlyHospital study, people with dementia and their relatives will be interviewed, which will allow us to gain to their perspective regarding a DFH as well. In addition, for our workshop, we recruited participants from only one network of dementia experts. In this network all professional dementia experts have a preexisting qualification as nurses and almost all of them work in the same hospital. The results of our workshop might have been different with a more heterogeneous sample related to hospitals and professional groups.

Regarding the methodological approach of the workshop, it should be considered that the same researchers who conducted the review also conducted the workshop, which may have influenced the discussion. Furthermore, the researchers did not permanently accompany the individual group work, so interesting contents of the discussion within the smaller working groups might have been not documented. In addition, the discussions of the results from the smaller working groups in plenary were not recorded but were protocolled by two people, which may also have influenced the results of the workshop. Another limitation arises from the questionnaires to prioritize the DFH characteristics and rate the feasibility of the subcategories, which were not pretested and could not be explained in detail due to time constraints of the one-day workshop. We were unable to include all the questionnaires in the analysis of prioritization because several of the characteristics were ranked the same. It is unclear whether the prioritization of the characteristics was fully understood or if these characteristics were considered equally important by the professional dementia experts. The small number of analyzable questionnaires may have an impact on the findings. However, our prioritization results indicate a clear trend.

## Conclusion

The involvement of professional dementia experts as stakeholders at the end of our review process allowed us to contextualize the review results within the hospital setting in Germany. Our findings clearly illustrate the relevance of the characteristics of a DFH as well as the gap between these and the current situation in hospitals. A perspective for future improvements could be the national dementia strategy [15] in Germany, which has highlighted the importance of dementia friendliness in hospitals. However, the involvement of professional dementia experts also showed that for the development of such a concept, people with dementia and their relatives need to be heard and involved in dementia care research. In addition, for future implementation of DFHs it is necessary to consider the available resources, funding options, influencing factors and the current situation and culture of hospitals and to address these with implementation strategies tailored to the organization.

### Supplementary Information

**Additional file 1.** Peer review report.

## Data Availability

Almost all data generated and analyzed in this study step (the workshop with professional dementia experts) would be available in an anonymous and aggregated way from the corresponding author upon reasonable request. For data requests, you can contact first author or central research data management (data-management-witten@dzne.de) at the DZNE site Witten.

## Abbreviations

ACTIVE: Authors and Consumers Together Impacting on eVidencE
DFH: Dementia-friendly hospital
DEMfriendlyHospital study: Characteristics of dementia-friendly hospitals study
