# Supplementary material for: Contextualizing the results of an integrative review on the characteristics of dementia-friendly hospitals: a workshop with professional dementia experts
Source: BMC Geriatr. 2023 Oct 19;23:678. doi: 10.1186/s12877-023-04312-3 (PMC10585930; doi:10.1186/s12877-023-04312-3)
Supplement: Supplementary file 1 — Additional file 1. Peer review report. [file 12877_2023_4312_MOESM1_ESM.docx]

<https://editorial.nature.com/submissions/92851af5-a7d4-4aa9-87b0-0c2bea1ce7fd/reviews-and-decisions/versions/1.1>

Claire M. C. O'connor 28-NOV-22

FEEDBACK FOR AUTHOR(S)

Thank you for the opportunity to review this paper that describes the outcomes from a workshop with hospital based nurses to evaluate the outcomes of a recent review around dementia friendly hospitals to contextualise the outcomes within the German hospital context. The study is interesting and important. My main concern is around the lack of inclusion of people with dementia and family carers. Some further suggestions to consider are outlined below. Many thanks.

Methods:

In the following sentence: “…were considered an intendent result.” Please clarify what is meant by this.

Figure 1, step 4, it should be spelt ‘questionnaire’.

Participants were asked to “discuss and rate the relevance of the assigned DFH characteristic for the patients with dementia, their relatives and the health care professionals”. I am wondering why only health care professionals were involved in this workshop. Including the voices of people with dementia and their relatives would be an important inclusion to ensure more meaningful outcomes for all stakeholders from this project. The importance of inclusion of these other stakeholders is illustrated clearly in the results when “the characteristic "valuing relatives" was rated as "somewhat relevant" for the patients.” I am sure that some people with dementia would rate “valuing relatives” as extremely important (if not vital) when in an unfamiliar and busy environment such as hospital. In the discussion, the authors identify that “the included descriptions of DFHs in our integrative review were primarily characterized by the perspective of professional dementia experts and health care practitioners”, which further indicates the importance of adding the voices of people living with dementia and family carers. This would create a much richer and more meaningful outcome to this study. Please consider running another workshop round involving people with dementia and family supporters, or provide a clear justification for why these other stakeholder groups were not included. I see now that this is mentioned in the limitations section. It is such an important inclusion to create rounded outcomes for this study; suggest adding clear reasoning upfront in the methods, and clarifying that these workshops with the professionals is just the first step in this process, and that the next phase will involve people with dementia and family carers.

Please provide more information about the anonymous questionnaire. How was it developed? Was it distributed during the workshop, or were participants asked to complete after the workshop?

Results:

For table 5, it is unclear what the numbers in the table represent. Are they number of respondents who selected that option for each question item? If so, if there were only 5 questionnaires that were analysed, how do some of these have bigger number, like 10, 7,8? Please clarify.

Discussion:

Only 5 questionnaires included in the analysis, which may have impacted on validity of the outcomes. This should be mentioned in the limitations.

FEEDBACK FOR EDITOR

Your recommendation

Revise

Is the study design appropriate to answer the research question (including the use of appropriate controls), and are the conclusions supported by the evidence presented?

No, but these points can be addressed with revisions

Comments: The voice of people with dementia is clearly missing from this study, however, it seems this paper may be the first step i the process and the authors may be preparing for this in a next step.

Are the methods sufficiently described to allow the study to be repeated?

Yes

Is the use of statistics and treatment of uncertainties appropriate?

I am not able to assess this

Is the presentation of the work clear?

Yes

Are the images in this manuscript (including electrophoretic gels and blots) free from apparent manipulation?

Not applicable

Confidential Comments to the Editor

Top of Form

Bottom of Form

Top of Form

How useful did you find this report?

Not useful    Extremely useful

Bottom of Form

<https://editorial.nature.com/submissions/92851af5-a7d4-4aa9-87b0-0c2bea1ce7fd/reviews-and-decisions/versions/2.0>

Bottom of Form

Steffen Heinrich 18-MAR-23

FEEDBACK FOR AUTHOR(S)

The topic of "dementia-friendly hospital" is relevant to the care of people with dementia and has not yet been conclusively investigated scientifically.

The project for this article sounds ambitious. However, the data collected so far is not sufficient for an in-depth understanding of the topic, as central target groups have not been included. These include, among others, people with dementia and their relatives. Since there will apparently be further data collection with the target group, this could improve the data situation.

In my opinion, however, this would still not be sufficient. Dementia is an interprofessional field and the hospital is an interprofessional setting. Why only nurses were used as experts to answer the research questions and not other disciplines such as doctors, physiotherapists or the social service remains unclear. Due to the chosen sample, there is a clear risk that the extracted results are biased systematically.

Some points of criticism are listed below:

In connection with the methodological approach (ACTIVE Framework), reference is made to an external source (Integrative Review) for further details (l. 90 -95). However, it would be appropriate to state the procedure for this empirical article in more than this rudimentary form.

The content analysis method used is only roughly described (l. 165f.).

The fact that only five of the 15 questionnaires out of a total of 16 could be evaluated in terms of prioritising DFH characteristics testifies to possibly massive difficulties in understanding the questionnaires by the participants. However, the motivation to complete the questionnaires seemed to be high with regard to the response rate. It remains unclear why two people did not provide information on their gender (Table 3). Furthermore, it is not clear from when to when and in which year the data collection took place.

Although global indications of barriers to implementing dementia-friendly processes in hospitals are given, it is not systematically explained why certain aspects of this overall process were described as good and others as less implementable (Table 6).

The fact that no pretesting of the questionnaires took place is not good scientific practice and unfortunately seems to have led to serious limitations in the quality of the results.

In the document there are the sections "Lessons Learned" and "Limitations". Here significant limitations of the methodology and data collection are described. This overall transparency is positive. However, when considering the limitations identified, it is surprising that an article was nevertheless prepared for publication instead of correcting the errors during the next planned data collection and merging them with the previous data. This would have provided the opportunity for a solid and clinically meaningful article. Thus, the increase in knowledge on this topic is limited and monodisciplinary, which does not really do justice to the topic.

See also: Handley M, Bunn F, Goodman C. Dementia-friendly interventions to improve the care of people living with dementia admitted to hospitals: a realist review. BMJ Open. 2017 Jul 16;7(7):e015257. doi: 10.1136/bmjopen-2016-015257. PMID: 28713073; PMCID: PMC5541590.

FEEDBACK FOR EDITOR

Your recommendation

Reject

Is the study design appropriate to answer the research question (including the use of appropriate controls), and are the conclusions supported by the evidence presented?

No, and there are fundamental issues that cannot be addressed

Comments: Due to the chosen sample, there is a clear risk that the extracted results are biased systematically.

Are the methods sufficiently described to allow the study to be repeated?

No, but these points can be addressed with revisions

Comments: Details are provided elsewhere but in the article itself, some key aspects regarding the methodology and methods are lacking (see comments).

Is the use of statistics and treatment of uncertainties appropriate?

Not relevant to this manuscript

Is the presentation of the work clear?

Yes

Are the images in this manuscript (including electrophoretic gels and blots) free from apparent manipulation?

Not applicable

Confidential Comments to the Editor

Top of Form

Bottom of Form

Top of Form

How useful did you find this report?

Not useful    Extremely useful

Bottom of Form

Claire M. C. O'connor 20-FEB-23

FEEDBACK FOR AUTHOR(S)

Thank you to the authors for addressing the reviewer feedback in the latest version of this manuscript.

FEEDBACK FOR EDITOR

Your recommendation

Accept

Is the study design appropriate to answer the research question (including the use of appropriate controls), and are the conclusions supported by the evidence presented?

Yes

Are the methods sufficiently described to allow the study to be repeated?

Yes

Is the use of statistics and treatment of uncertainties appropriate?

I am not able to assess this

Is the presentation of the work clear?

Yes

Are the images in this manuscript (including electrophoretic gels and blots) free from apparent manipulation?

Not applicable

Confidential Comments to the Editor

Version 3 (final)

<https://editorial.nature.com/submissions/92851af5-a7d4-4aa9-87b0-0c2bea1ce7fd/reviews-and-decisions/versions/3.1>

Bottom of Form

Steffen Heinrich 30-MAY-23

FEEDBACK FOR AUTHOR(S)

I have read the responses to my comments with interest. There are still some clear limitations in the methodology. Some quality assurance measures that were not implemented are difficult to explain (e.g. no pretest of the questionnaire). The majority of the limitations are dealt with transparently, which is a central strength of the article in the sense of "lessons learned". Thus, despite the shortcomings, the article can make a relevant contribution to contextualizing the results of an integrative review by experts and what should be considered (or avoided) in the process.

These engagement processes are very important and have been described only to a limited extent in existing literature so far. Publication of the article in its current form (with its limitations that cannot be readjusted further) can therefore still be justified. It will also be important to see what further findings the project will produce and how these will be published in conjunction with this article in order to address central aspects such as interprofessionality in the setting. Only time will tell. However, as the authors rightly point out, a detailed and interprofessional development process towards the planned framework could then be shown. Should this succeed, the planned framework would have the requirements to practically address the needs and requirements of people with dementia in hospitals. In this regard, I would like to emphasise very urgently: A framework for care practice is only as good as it can actually be applied in practice. It seems to me that the authors are clearly addressing this aspect in their further project planning. Good luck for the further course of your project.

FEEDBACK FOR EDITOR

Your recommendation

Accept

Is the study design appropriate to answer the research question (including the use of appropriate controls), and are the conclusions supported by the evidence presented?

No, but these points can be addressed with revisions

Are the methods sufficiently described to allow the study to be repeated?

Yes

Is the use of statistics and treatment of uncertainties appropriate?

Not relevant to this manuscript

Is the presentation of the work clear?

Yes

Are the images in this manuscript (including electrophoretic gels and blots) free from apparent manipulation?

Yes

Confidential Comments to the Editor

In its current form, there are central limitations that can no longer be eliminated (especially the sample and the survey instrument). I have evaluated this article with reference to further project planning. It will only be decided in the next few years whether the value of the article should be assessed as low or high (for details, please see my reviewer comments).

© 2023 Springer Nature
